# Co-existence of multiple strains of porcine circovirus type 2 in the same pig from China

**DOI:** 10.1186/1743-422X-8-517

**Published:** 2011-11-13

**Authors:** Shao-Lun Zhai, Sheng-Nan Chen, Zu-Zhang Wei, Jian-Wu Zhang, Lv Huang, Tao Lin, Cheng Yue, Duo-Liang Ran, Shi-Shan Yuan, Wen-Kang Wei, Jin-Xue Long

**Affiliations:** 1 Department of Swine Infectious Diseases, Shanghai Veterinary Research Institute, Chinese Academy of Agricultural Sciences, Shanghai 200241, China; 2 Institute of Veterinary Medicine, Guangdong Academy of Agricultural Sciences, Guangzhou 510640, China; 3 College of Veterinary Medicine, Xinjiang Agricultural University, Urumqi 830052, China

**Keywords:** Porcine circovirus type 2, Multiple viral strains, Co-existence, New infection form

## Abstract

Pigs are often co-infected by different viral strains from the same virus. Up to now, there are few reports about co-existence of different porcine circovirus type 2 (PCV2) strains in China. The aim of this study was to evaluate it in Chinese swine herds. 118 PCV2 positive DNAs isolated from diseased pigs identified by classic PCR were re-detected using a modified differential PCR assay. The results indicated that co-existence rates of PCV2 were 32.2% (38/118) in diseased pigs and 0% (0/41) in asymptomatic pigs. Four PCV2 complete genomes were cloned from two co-infected samples and their nucleotide (nt) identities were 95%-97.3%. The phylogenetic analysis showed that four PCV2 strains were divided into different genotypes, PCV2a, PCV2b, PCV2d and PCV2e, respectively. In addition, co-existence were not detected in 41 serum samples from healthy pigs but PCV2 single infection (31.7%, 13/41) existed. These data revealed that the co-existence of different strains of PCV2 might contribute to the development of more severe clinical symptoms for pigs. This is the first report confirming the co-existence of different PCV2 strains in Chinese swine herds. Meanwhile, this study could help us to understand new infection and prevalence forms of PCV2 clinically.

## Findings

Porcine circovirus type 2 (PCV2), a small but powerful virus, is the major etiological agent of porcine circovirus associated diseases (PCVAD), which is involved in postweaning multisystemic wasting syndrome (PMWS), porcine dermatitis and nephropathy syndrome (PDNS), porcine respiratory disease complex (PRDC), congenital tremors type II (CT), reproductive failure [1-5].

PCV2 belongs to a member of the genus *Circovirus *of the family *Circoviridae*, which possesses a small, circular, single-stranded DNA genome (1767/1768 bp) [2]. According to the present definition of PCV2 genotypes, PCV2 are divided into five genotypes (PCV2a, PCV2b, PCV2c, PCV2d, PCV2e) [6,7], and their genomic nucleotide (nt) sequences differ by approximately 5%. The circular genome of PCV2 encodes two major open reading frames (ORF): ORF1 (945 bp) encodes the replicase protein involved in viral replication [8], ORF2 (702/705 bp) encodes the immunogenic capsid protein [6,7,9]. PCV2a and PCV2b have been reported in Europe and Asia, but PCV2b was not detected in North America prior to the year of 2004. In 2005, PCVAD outbroke in Canada [10,11] and in the United States [12], respectively. An emerging genotype, PCV2b, was considered as an important pathogen of that murrain. Since 2008, a third genotype (PCV2c) was reported in Denmark with only three sequences to date [6,13]. However, in China, in addition to PCV2a and PCV2b prevalence, another two genotypes (PCV2d and PCV2e) were also detected [7].

At present, PCV2 were frequently detected in Chinese swine herds, but no data reported on the co-existence of different PCV2 strains. Therefore, the goal of this study was to obtain information about the PCV2 co-existence in Chinese swine herds.

118 PCV2 positive DNAs isolated from clinical diseased pigs suffering from PMWS and PRDC between 2008 and 2009 were identified by classic PCR and stored at -30°C freezer until use. Moreover, to detect whether co-existence of PCV2 occurred in healthy pigs (about 60-day old) or not, 41 DNAs were isolated from 41 serum samples in two healthy pig farms of the Zhejiang province. Due to the same signature motif for PCV2a and PCV2e and the same/similar signature motif for PCV2b, PCV2c and PCV2d, the previous differential PCR assay [14] was minor modified, which was involved in the design of one communal upstream primer and two different downstream primers. In this study, the detection primers for PCV2a and PCV2e were F-PCV2ab (5'-CAGTTCGTCACCCTTTCCC-3') and R-PCV2a (5'-GGGGGACCAACAAAATCTC-3'), while the detection primers for PCV2b, PCV2c and PCV2d were F-PCV2ab and R-PCV2b (5'-GGGGCTCAAACCCCCKCWC-3'). PCV2 differential PCR reactions were performed on each sample in a 25 μl reaction mixture, which contained 0.5 μl of μM upstream primer, 0.5 μl of μM downstream primer, 12.5 μl of 2 × master mix (containing 0.1 U Taq DNA Polymerase μl^-1^, 0.4 mM each dNTPs, 2 × Taq buffer) (Lifefeng Inc., Shanghai, China), 8.5 μl of sterile water, and 3 μl of sample DNA. DNA amplification was initiated by preheating for 5 min at 94°C, followed by 40 cycles at 94°C for 15 s, 60°C for 20 s, and 72°C for 30 s, and a final extension for 10 min at 72°C.

To determine the complete genomic sequences from co-infected samples, primers (F-PCV2SacII and R-PCV2SacII) were used as described previously [15] to amplify the complete genome of all PCV2 genotypes. The PCR products, approximate 1.76 kb, were purified by BioDev Gel Extraction System A kit (Beijing, China) and ligated into vecter pBST-II (TIANGEN Inc., Beijing, China). The positive pBST-PCV2 recombination plasmids were sequenced by T7, T3 and PCF1096 (5'-GGATATTGTAKTCCTGGTCG-3') primers, respectively. Considering having difficulty in obtaining sequenes of different PCV2 genotypes from the same PCR product, initially, only partial gene of open reading frame 2 (ORF2) was sequenced by using primer PCF1096 for those positive recombination plasmids. When genome sequences of ORF2 were different from each other, plasmids were then sequenced by T7 and T3 primers. Sequences generated in this study were deposited in GenBank (accession no. GQ359003, GQ359004, GU001709 and GU001710), meanwhile, their nt identities were determined using the Clustal W alignment program (DNASTAR software). Phylogenitic tree based on neighbour-joining methods was constructed by MAGE version 4.0. Recombination event was evaluated by recombination detection program (RDP) 3.34 software.

118 PCV2 positive DNAs were identified by differential PCR assay, and the results showed that 36 samples contained PCV2a/2e-single infection, 44 samples contained PCV2b/2c/2d-single infection, and 38 samples (32.2%) were positive for co-existence. However, co-existence were not detected in 41 samples from healthy pigs but PCV2b/2c/2d-single infection (13/41) occurred.

**Figure 1:**
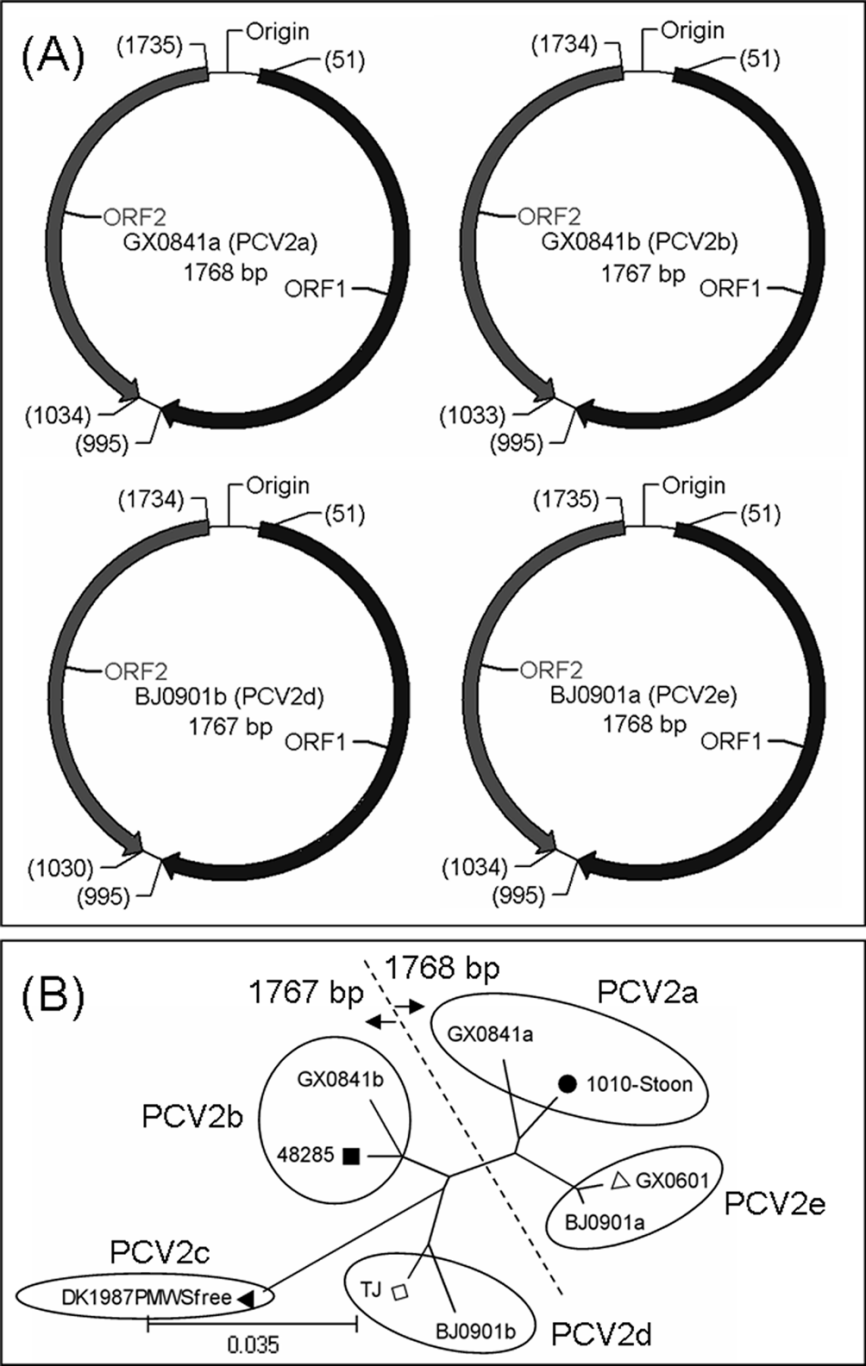
**Genome organization (A) and phylogenetic analysis (B) of PCV2 strains generated in this study**. Note: PCV2a reference strain 1010-Stoon (Accession No. AF055392) labeled with '•', PCV2b reference strain 48285 (Accession No. AF055394) labeled with '■', PCV2c reference strain DK1987PMWSfree (Accession No. EU148504) labeled with '▲', PCV2d reference strain TJ (Accession No. AY181946) labeled with '□', PCV2e reference strain GX0601 (Accession No. EF524532) labeled with 'Δ'.

**Figure 2:**
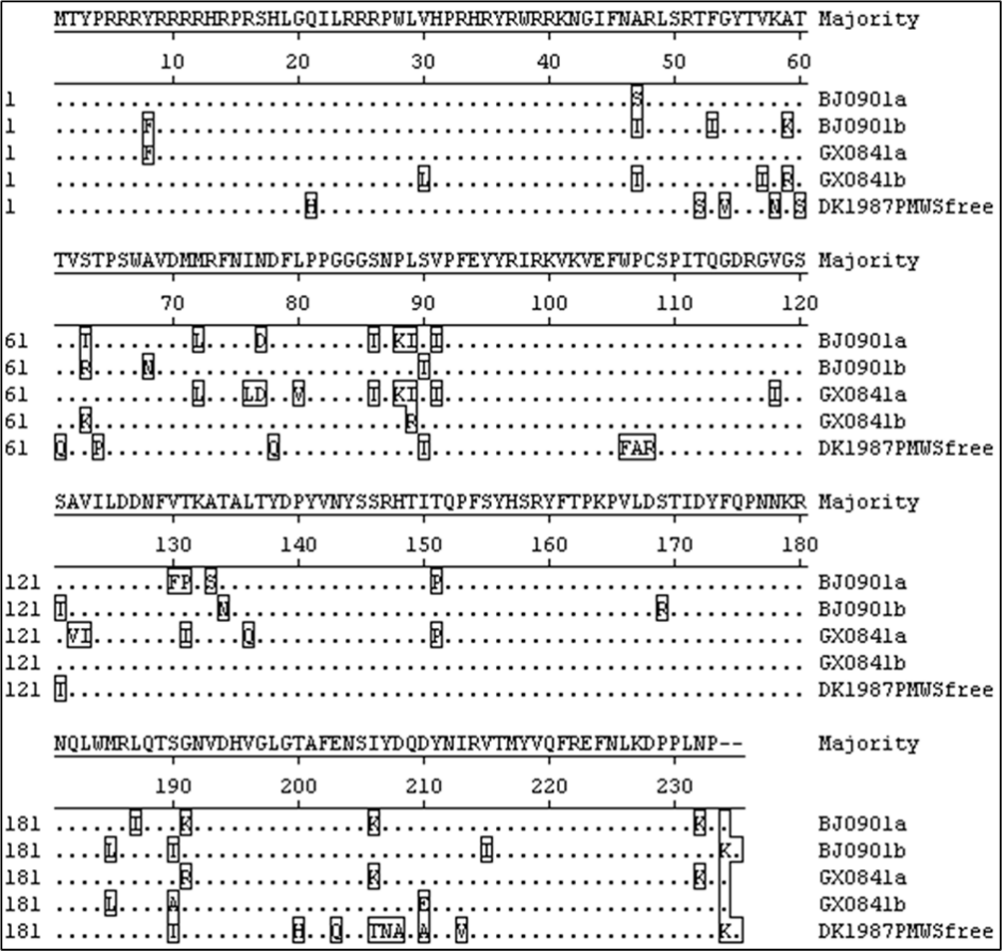
**Sequence alignment results about coding aa sequences of capsid gene (ORF2)**.

To primarily determine co-existence types of PCV2, the differential PCR products were submitted to sequencing company (HuaDa Gene) and directly sequenced using F-PCV2ab primer. Sequencing results of 38 co-infected samples showed that co-existence genotypes were PCV2a-PCV2b (12/38), PCV2a-PCV2d (15/38) and PCV2e-PCV2d (11/38), respectively. In addition, 13 PCV2b/2d PCR products from healthy serum samples were also sequenced and PCV2 genotypes were identified as PCV2b (5/13) and PCV2d (8/13). Interestingly to us, PCV2c genotype was not detected in this study. To further confirm the co-existence of different PCV2 genotypes, four full-length clones (named as GX0841a, GX0841b, BJ0901a and BJ0901b) were obtained from the samples of GX0841 and BJ0901. Sequence identities were 95%-97.3% among them (Table 1). GX0841a and BJ0901a strains had the same genome organizations (ORF1: from nt 51 to nt 995; ORF2: from nt 1735 to nt 1034), while GX0841b and BJ0901b had distinct genome structure, (ORF1: from nt 51 to nt 995; ORF2: from nt 1734 to nt 1033) and (ORF1: from nt 51 to nt 995; ORF2: from nt 1734 to nt 1030), respectively. In other words, their differences mainly lay in the position and size (702 bp for GX0841a, GX0841b and BJ0901a; 705 bp for BJ0901b) of ORF2 in the whole genome (Figure 1A). Phylogenetic analysis based on the complete genome showed four strains were divided into four different genotypes, PCV2a (GX0841a), PCV2b (GX0841b), PCV2d (BJ0901b) and PCV2e (BJ0901a) (Figure 1B). Moreover, we also evaluated their identities of nt and amino acids (aa) among ORF1, ORF2 and ORF3, the sequence alignment results showed that the identities of ORF2 (87.3%-94.6% for nt and 83.3-93.6% for aa) (Figure 2) were lower than ORF1 (97%-99% for nt and 98.7%-99.7% for aa) and ORF3 (97.5%-98.7% for nt and 91.4%-95.2% for aa) (data not shown). In addition, we could not find significant recombination breakpoints from different PCV2 genotypes isolated co-infected samples by sequence alignment and RDP (data not shown).

**Table 1:** Pairwise percentage nucleotide identity comparisons of PCV2 complete genome

Isolate	Identity (%) with				
	**GX0841a** **(PCV2a)**	**GX0841b** **(PCV2b)**	**BJ0901a** **(PCV2e)**	**BJ0901b** **(PCV2d)**	**DK1987PMWSfree** **(PCV2c)**

GX0841a (PCV2a)	100	95.4	97.3	95.0	93.8
GX0841b (PCV2b)	_	100	95.5	95.9	94.6
BJ0901a (PCV2e)	_	_	100	95.1	94.2
BJ0901b (PCV2d)	_	_	_	100	94.6
DK1987PMWSfree (PCV2c)	_	_	_	_	100

Differential PCR was developed for the first time by Hesse et al. [14], at that time, they designed primers only to detect PCV2a and PCV2b not PCV2c and PCV2d. In this study, co-existence rates were (32.2%, 38/118), which was much higher than that 25% (24/97) in their study (p < 0.05, χ2 test). The reason might be that differential PCR was modified and optimized, which could increase PCV2 detection rates (such as new genotypes: PCV2c and PCV2d). In the present study, there was PCV2b- or PCV2d- single infection detected in healthy pigs, which was the similar to the first report on PCV2 infection in a high-health Canadian swine farm [1]. The above data indicated that pigs co-infected by PCV2 multiple genotypes or strains displayed more severe lesions, which was similar to the previous study [16]. However, Opriessnig et al. [17] confirmed there was no significant difference in virulence between PCV2a and PCV2b but prior to infection provides heterologous protection. This might imply the co-existence mechanism of multiple PCV2 strains is confusing.

Although four PCV2 genotypes (2a, 2b, 2d, 2e) have been determined in Chinese herds in the previous study [7], however, in the present study, it is the first time confirmed that the co-existences of PCV2a-PCV2b, PCV2a-PCV2d and PCV2d-PCV2e. PCV2c, as a distinct genotype, was only reported in Denmark rather than other countries and regions [6,13]. In this study, it was not still detected in Chinese swine herds, which was similar to the results in the previous study [7]. To some extent, we could think that there were different PCV2 genotypes on different pig farms, different regions, and even different time [13]. Isolations of two PCV2 full-length clones from the same sample was a time-consuming process. Therefore, it should be necessary for us to design genotype-specific primers for amplifying the complete genomes of different genotypes in the future.

PCV2 mainly evolves by mutation and/or recombination. In comparison to GX0601 strain from 2006 in China, BJ0901a strain from 2009 in China had 12 nt substitution, while, in comparison to TJ strain from 2002 in China, BJ0901b strain from 2009 in China had 31 nt substitution. In this study, BJ0901a (4 × 10^-3^) and BJ0901b (4.4 × 10^-3^) had higher nt substitution rates than the estimated (1.2 × 10^-3 ^substitutions/site/year) by Firth et al. [18]. Co-existence of different PCV2 genotypes in the same pig could contribute to PCV2 recombination. There were no significant recombination breakpoints detected in this study, although putative recombination breakpoints were identified previously [14,19-23]. Here, some recombination events were questioned, due to possible artifacts. In fact, the bulk of PCV2 complete genome deposited in GenBank have been obtained by PCR amplifying using multiple primers from PCV2 genome conserve regions. In this study, the full-length of PCV2 from co-infected samples were amplified by PCR using a pair of primers, which could avoid artificial recombination events generating. In addition, nt sequencing of complete PCV2 genome obtained through rolling-circle amplification assay could overcome this problem [24,25].

In conclusion, this is the first time demonstrating co-existence of different PCV2 genotypes or strains in China. This study could help us to better understand molecular epidemiology and new infection forms of PCV2. Modified differential PCR method saved some time than before, which could fasten the detection process of PCV2 multiple genotypes clinically. Moreover, the presence of multiple genotypes in the same pig should be taken into consideration when obtaining molecular epidemiology of PCV2. Co-existence mechanism of multiple PCV2 strains or genotypes should have been elucidated through more studies.

## Competing interests

The authors declare that they have no competing interests.

## Authors' contributions

SLZ, SNC, ZZW, JWZ, LH and TL obtained samples and extracted viral DNA. SLZ carried out the PCR and sequencing studies and drafted the manuscript. SNC, CY and DLR revided the manuscript. SSY, JXL and WKW conceived the study and reviewed the manuscript. All authors read and approved the final manuscript.
